# Analytic hierarchy process: An innovative technique for culturally tailoring evidence‐based interventions to reduce health disparities

**DOI:** 10.1111/hex.13022

**Published:** 2020-01-07

**Authors:** Jaime A. Corvin, Isabella Chan, Claudia X. Aguado Loi, Ian Dollman, Junius Gonzales

**Affiliations:** ^1^ Department of Global Health College of Public Health University of South Florida Tampa FL USA; ^2^ Department of Health Sciences and Human Performance University of Tampa Tampa FL USA; ^3^ New York Institute of Technology New York NY USA

**Keywords:** chronic disease, depression, disparity, Latino, patient driven

## Abstract

Latinos in the United States represent a disproportionate burden of illness and disease and face barriers to accessing health care and related resources. Culturally tailored, evidence‐based interventions hold promise in addressing many of these challenges. Yet, ensuring patient voice is vital in the successful development and implementation of such interventions. Thus, this paper examines the application of analytic hierarchy process (AHP) to inform the augmentation and implementation of an evidence‐based chronic disease self‐management programme for underserved Latinos living with both minor depression and chronic illness. The process of AHP allows for direct input from the individuals that would utilize such a programme, including afflicted individuals, their family members and the health educators/promotores that would be responsible for implementation. Specifically, 45 participants, including 15 individuals with chronic disease, 15 family members/caregivers and 15 promotores, partook in the Stakeholder Values Questionnaire, which elicited preferences and values regarding major goals, processes and content for the intervention. AHP was employed to analyse pairwise comparison ratings and to determine differences and similarities across stakeholder groups. This analytical technique allowed for the adaptation of the EBI to stakeholders' specific priorities and preferences and facilitated complex decision‐making. Findings not only shed light on similarities and differences between stakeholder groups, but also the magnitude of these priorities and preferences and allowed the intervention to be driven by the participants, themselves. Applying AHP was a unique opportunity to optimize the decision‐making process to inform cultural adaptation of an EBI while considering multiple viewpoints systematically.

## 1 INTRODUCTION

The 2012 National Healthcare Disparities Report asserts that, despite efforts to enhance access to care, access has not improved among most racial and ethnic groups.^1^ In fact, racial and ethnic minorities fare worse in terms of access to health care and associated outcomes as compared to their white counterparts.^2^ Among Latinos, the largest minority population in the United States, contributing factors to poor health outcomes include lack of access to and utilization of preventive care, lack of health insurance and linguistic and cultural barriers.^3^, ^4^ Further, Latinos continue to face a disproportionate burden of illness and disease. In fact, diabetes rates among Latinos are nearly double that of non‐Hispanic whites.^4^

Culturally tailored, evidence‐based interventions (EBI) hold much promise in overcoming these challenges.^5^ EBIs such as Tomando Control de su Salud, a chronic disease self‐management programme developed by Stanford University, have been touted for their ability to enhance chronic disease self‐management practices, including improvements in health behaviours, health status, enhanced self‐efficacy and fewer emergency room visits.^6^ Such programmes have been disseminated globally to diverse populations and have shown positive results.^7^ Chronic disease self‐management programmes (CDSMP) have several features that make them worthy of adaptation, particularly the format used to provide health education, the utilization of peer group members and lay leaders, and the use of multidimensional techniques to address nutrition, physical activity, problem‐solving, sleep, fatigue and patient empowerment through the enhancement of self‐efficacy and positive behaviour change.^8^, ^9^

Yet, questions remain over how to ensure that EBIs are culturally tailored to local needs. The complex interplay between chronic illness and the host of factors that impact access to and utilization of health services by underserved Latinos requires EBIs to be responsive to local situation reality faced by Latinos. The adaptation of such interventions is costly and time‐consuming and requires considerable resources. One approach to guiding adaptation includes seeking input from the drivers or potential end‐users of the programme, an approach often employed in consumer marketing, new product development and assessing business risk levels.^10^ Analytic hierarchy processing (AHP), a technique developed by Thomas Saaty,^11^, ^12^ is one such approach, allowing the human drivers and key end‐users to guide primary decision‐making. AHP has been shown to be effective in guiding multi‐attribute decision‐making, and the process allows decision‐makers to model complex problems using a hierarchical structure. AHP is used to prioritize criterion, in this case programme objectives and content. The resulting prioritization ranks items within the model ratio scale where priorities or weights are derived for each objective or subobjective, allowing the researcher to select the objectives that will have the most impact and helping to guide ‘best fit’ decision‐making.

### 1.1 Value of analytic hierarchy process

AHP is a ‘science of scaling based on math, philosophy and psychology’ in which a complex decision is broken down into factors that are arranged by the researcher in an ordered structure to allow weights to be assigned to each factor.^11^ Rather than focusing on a single criterion, AHP takes into account all of the applicable criteria concurrently, encompassing a more systematic and transparent approach.^13^ The decision‐makers (in our case those for which the programme was being tailored) were asked pairwise comparison questions, deciding the importance of one criterion relative to another.^13^

Traditionally, AHP has been used in the field of business as a technical and managerial group decision‐making process where one seeks to find the partialities of differing groups from a macro‐level view.^14^ The value of AHP is its flexibility and ability to be precisely customized to each individual challenge.^10^ In addition to product screening and development, those in the business sector also employ AHP as a tool for determining cost‐effectiveness and how to appropriately allocate finite resources.^13^ AHP successfully allows complex decisions to be more easily made with consideration of multiple criteria.

With the effectiveness and value of AHP evident, it is reasonable to establish that it can be translated to complex issues related to health programme decision‐making as well. AHP has been used in health‐related fields to assess patient satisfaction in services, determine liver transplantation patient priority setting, understand performance of intensive care units, accompany geographical information system (GIS) data in understanding the health needs of communities, assess applicability of telehealth programmes and help patients decide the specific course of treatment that best suits their needs.^15^, ^16^, ^17^, ^18^, ^19^, ^20^, ^21^, ^22^, ^23^, ^24^ Groups are calling for the engagement of patients in research, including patient voice in research, and patient centred health care.^25^, ^26^, ^27^ AHP is gaining attention as useful methodology to engage patients. However, application of AHP to the development of health promotion or health education interventions or in tailoring health education models to the needs of beneficiaries of the programme is scarce.^28^ Thus, AHP as a tool to customize the objectives and content prioritization of an existing evidence‐based programme to a specific target population would be of immense benefit to all stakeholders involved.

Before launching any health promotion campaign, it is imperative to ensure the relevance of the programme and feasibility of adaptation among the target population. Thus, the multiphase parent study of this paper^19^ sought to adapt Stanford University's CDSMP, Tomando Control de su Salud (Tomando), to the needs and preferences of underserved Latinos in the Tampa Bay area suffering from both minor depression and a chronic illness and to determine whether the adapted intervention would be suitable for the community. This paper discusses Phase II of the parent study, which elicited preferences and values from key end‐users for major goals, processes and content of Tomando using Stakeholder Values Questionnaire and AHP.

## 2 METHODS

This study employed AHP to inform the development and implementation of an EBI to enhance chronic disease self‐management among underserved Latinos living with both minor depression and chronic illness. The approach built upon initial, formative research findings,^29^ which assessed barriers and facilitators to chronic disease self‐management, ultimately allowing for a robust assessment of needs, preferences and priorities among the target population and the implementation of an intervention driven almost entirely by the population it intended to serve. University institutional review board approval was sought from the research institution prior to implementation of the study (#107512).

### 2.1 Sample population

Almost 30% of the population of Hillsborough County, the area of focus for this study, identify as Hispanic/Latino.^30^ This population, now the largest minority population in both the county and the United States, lacks access to resources and services, particularly intensive, comprehensive and specialized services, and faces linguistic and cultural barriers to accessing care.^4^ To help fill this gap, this study targeted underserved Latinos living with a chronic illness and minor depression, noted in this study as individuals with chronic disease (ICDs), their family members (FMs) and the promotoras (P) who would be responsible for delivering the intervention. Fliers at local community outreach events, the local library, clinics and at community partner sites were used to recruit participants. Eligibility requirements for ICDs included the following: (a) minor depression as measured by the Patient Health Questionnaire two‐item screener (PHQ‐2)^31^; (b) self‐reported diagnosis of hypertension, diabetes or cardiovascular disease; (c) residence in Hillsborough county, Florida; and (d) self‐identification as Hispanic/Latino. Family member participants were nominated by ICDs as being active in their care. To be eligible for participation as a promotoras, participants had to be currently working as a health promotor, lay health educator or Promotoro/a in Hillsborough county. Promotoras were specifically recruited through local organizations delivering a variety of health programmes and services. None of the promotoras recruited had any experience with the Tomando programme and were not associated with the research project. All participants were recruited specifically for this phase of the research, and there was no overlap among participants from this phase of the study and Phase I of the parent study.

### 2.2 Development of the Stakeholder Values Questionnaire

Findings from qualitative data collected through Phase I semi‐structured interviews (n = 37) and structured surveys (n = 35) with promotoras, nurses, physicians and CBO leaders, and focus groups (N = 9; n = 42) with ICDs and FMs were used to guide the development of our Stakeholder Values Questionnaire.^29^ While findings from this phase are published elsewhere, this Stakeholder Values Questionnaire was developed based on emergent themes, key priorities and needs which arose from the formative research stage.^29^, ^32^ This included challenges with managing chronic illness, unmet needs and the importance of support and education for those living with chronic illness. Additionally, the questionnaire was designed to also evaluate the core elements of the Tomando programme and to elicit preferences and values from stakeholders regarding major goals, processes and content for the intervention.^33^ Specifically, the questionnaire evaluated the important elements of the intervention (ie skill‐building or informational and educational materials), the structure of the programme (ie the number of sessions and the content of those sessions), who should lead the programme (ie medical professionals, promotoras), and attendance (ie either alone or with a partner) at the programme. The questionnaire was developed using the AHP method and presented respondents with a series of paired comparisons for each objective and subobjective for their input.

### 2.3 Data collection

Prior to completing the questionnaire, 45 participants, 15 from each key stakeholder group (ie ICDs, FMs and promotoras/outreach workers) received a presentation on Tomando by trained research staff and watched a video developed to provide additional in‐depth information about the programme. Participants were then asked to offer input on the Tomando programme and provide guidance on priority needs and preferences through the Stakeholder Values Questionnaire. Each questionnaire was orally administered in Spanish by a member of the research team, which allowed participants to select between paired comparisons, while also assigning a weight to their selection. A sliding scale tool was used to allow participants to select their response and the desired weight. Questionnaires took 10‐20 minutes to complete, and participants received a $40 stipend for their participation.

### 2.4 Data analysis

Following data collection, participant decisions were entered directly into Expert Choice ©,^34^ a software programme designed to facilitate and analyse choices through the collaborative decision‐making process. AHP was employed to analyse stakeholders' pairwise comparison ratings and to determine differences and similarities given by the three stakeholder groups. Key priorities regarding the refinement of the educational programme as well as differences and similarities across stakeholder groups were analysed and ranked. This ranking highlighted the differences between the stakeholder groups and allowed the research team to tailor the new educational health intervention to their specific priorities and preferences.

## 3 RESULTS

### 3.1 Participant demographics

The mean age for the total population was 49.8 years, with ICDs being older, at an average of 56.5 years compared to 45.3 years for FMs and 47.7 years for promotoras. The majority of participants (91.1%) reported having a high school diploma, GED, vocational school training or some college. Among promotoras, three individuals (20%) had a graduate degree. The majority of the population (64.4%) was married or living with a partner, and the average household income was between $10 000 and $39 999, with some variability between samples. Hypertension (20%) and diabetes (40%) were the most commonly reported illnesses among ICDs. While not a criterion for enrolment, 40.1% of FMs and 40% of promotoras also reported a chronic illness. Furthermore, over 40% of participants reported being uninsured. The vast majority of participants, 62.2%, reported preferring to receive their health‐care information in Spanish. Detailed participant demographics are presented in Table 1.

**Table 1:** Participant demographics

	Total N = 45		ICD n = 15		FM n = 15		Promotores n = 15	
Mean Age (SD)			56.5	13.4	45.3	17.7	47.7	11.2

	n	%	n	%	n	%	n	%
Gender								
Males	9	20	6	40	2	13.3	1	6.7
Females	36	80	9	60	13	86.7	14	93.3
Education								
Less than high school	1	2	—	—	1	6.7	—	—
High school graduate or GED	14	31	7	46.7	4	26.7	3	20
Vocational school or college	27	60	8	43.3	10	66.7	9	60
Graduate School/Professional	3	7	—	—	—	—	3	20
Marital Status								
Married/cohabiting	29	64	11	73.3	10	66.7	8	53.3
Single, never married	5	11	1	6.7	3	20	1	6.7
Widowed	3	7	1	6.7	1	6.7	1	6.7
Separated/divorced	8	18	2	13.3	1	6.7	5	33.3
Income								
<$10 000	8	18	5	33.3	2	13.3	1	6.7
$10 000‐$39 999	29	64	10	66.7	10	66.7	9	60
$40 000‐$79 999	7	16	—	—	2	13.3	5	33.3
>79 999	1	2	—	—	1	6.7	—	—
Country of origin								
Foreign born	35	78	13	86.7	13	86.7	9	60
US born	10	22	2	13.3	2	13.3	6	40
Time in the USA								
6 y or less	4	9	—	—	1	6.7	3	20
Between 6 and 25 y	24	53	10	66.7	9	60	5	33.3
More than 25 y	9	20	4	26.7	3	20	2	13.3
US born	9	20	1	6.7	2	13.3	6	40
Chronic illness								
Cardiovascular disease	4	9	3	20	—	—	1	6.7
Diabetes	8	18	6	40	1	6.7	1	6.7
Hypertension	5	11	3	20	1	6.7	1	6.7
Other	13	29	6	40	4	26.7	3	20
Health insurance								
No	18	40	6	40	10	66.7	2	13.3
Yes	27	60	9	60	5	33.3	13	86.7
English speaking ability								
Not at all	3	7	2	13.3	1	6.7	—	—
Not well	18	40	6	40	9	60	3	20
Well	23	51	6	40	5	33.3	12	80
Language preference								
English	9	20	1	6.7	2	13.3	6	40
Spanish	28	62	12	80	12	80	4	26.7
Both	8	18	2	13.3	1	6.7	5	33.3
Employment status								
Full‐time	20	44	5	33.3	6	40	9	60
Part‐time	10	22	2	13.3	3	20	5	33.3
Retired	4	9	3	20	1	6.7	—	—
Homemaker	4	9	1	6.7	3	20	—	—
Unemployed	7	16	4	26.7	2	13.3	1	6.7

Overall, participants were asked questions regarding important elements for a CDSMP intervention. The Stakeholder Values Questionnaire was divided into three sections: (a) important elements of an intervention, (b) priming participants for an intervention and (c) sustaining positive outcomes. Critical findings from each area are discussed in detail below. Rank scores for stakeholder groups are noted as C for the combined model, P for promotoras, ICD for individuals with chronic disease and FM for family.

### 3.2 Identifying important elements of the intervention

#### 3.2.1 Content assessment

**Figure 1:**
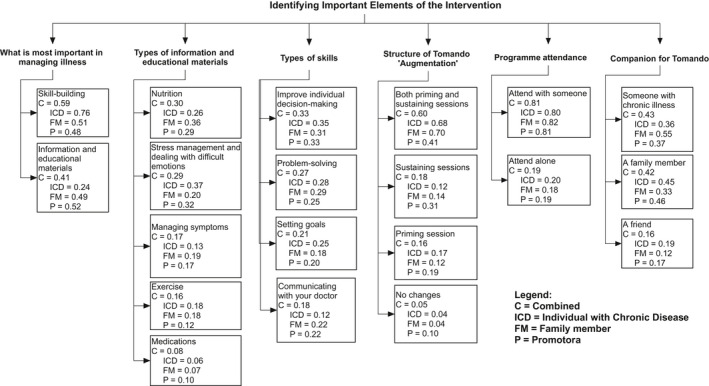
Identifying important elements of the intervention

One of the most vital aspects of tailoring this intervention was determining priorities in managing illness, including ensuring appropriate content and strategies for sharing needed information. Accordingly, participants answered questions regarding the importance of skill‐building and educational materials in improving health and enhancing the management of chronic illness. In the combined model (C), which included all stakeholder groups, enhancing skill‐building was ranked first (C = 0.59) compared to educational‐ and informational‐based elements (C = 0.41) (see Figure 1).

However, variations between group models were noted. Rank orderings differed in the promotoras model, which ranked educational components first (P = 0.52), followed closely by skill‐building (P = 0.48). Both ICDs and FMs placed more weight on skill‐building (ICDs = 0.76 and FM = 0.51), with ICDs placing greater importance on this element than on information and education (ICD = 0.24). To delve further into the content required for either skill‐building or educational sessions, each area was probed individually.

#### 3.2.2 Educational components

Educational components identified in the formative research stage as being important to self‐management, including nutrition and stress management, were analysed. Components that already existed through the Tomando programme, such as medication management, were also analysed for appropriateness. In the combined model, information on nutrition was ranked first (C = 0.30), followed closely by stress management (C = 0.29), then managing symptoms (C = 0.17), exercise (C = 0.16) and information regarding medications and their usage (C = 0.08). However, variation across models was noted. The FM model placed considerably more weight on nutrition (FM = 0.36) compared to stress management and dealing with difficult emotions (FM = 0.20). However, in the ICD model, stress management and dealing with difficult emotions were ranked as the most important element (ICD = 0.37), followed by nutrition (ICD = 0.26). Similarly, promotoras ranked stress management first (P = 0.32), followed closely followed by nutrition (P = 0.29). Educational information regarding medications ranked lowest across all three groups overall (C = 0.08).

#### 3.2.3 Types of skills

Participants were also asked about specific strategies for enhancing skill‐building, including improving individual decision‐making, problem‐solving, goal setting and communicating with a doctor. The combined model ranked enhancing individual decision‐making first (C = 0.33), followed by problem‐solving (C = 0.27), improving goal setting (C = 0.21) and learning to better communicate with your doctor (C = 0.18). While general trends were consistent across the three models, interesting differences were noted. For example, the ICD model ranked learning to better communicate with your doctor significantly less important than other options (ICD = 0.12) when compared to the FM (FM = 0.22) and promotora (P = 0.22) models.

#### 3.2.4 Assessing structure

An important consideration of any intervention is its structure. Without an amenable structure, participants may not attend nor benefit from the content presented. Therefore, a portion of the questionnaire focused on designing a structure for Tomando that met the needs of participants, particularly concerning the number of sessions and the timing of those sessions.

The combined model ranked the incorporation of both priming and sustaining sessions to Tomando as the preferred ‘augmentation’. Consensus across groups was demonstrated, and adding both priming and sustaining sessions was the clear choice among ICDs (ICD = 0.68), FMs (FM = 0.70) and promotoras (P = 0.41), with other options ranked considerably lower. Interestingly, ICDs ranked having a priming session as their second choice (ICD = 0.17) and a sustaining session as their third choice (ICD = 0.12), while FMs ranked sustaining sessions as their second choice (FM = 0.14) and priming sessions (FM = 0.12) as third choice. Promotoras demonstrated a similar trend to FMs, ranking the incorporation of both priming and sustaining sessions as the preferred option, followed by adding a sustaining session (P = 0.31), then adding a priming session (P = 0.19) and finally making no changes to Tomando (P = 0.10).

#### 3.2.5 Attending with a partner

There was consensus across groups that attending the programme with a companion was optimal. However, there was variation between groups regarding who would be the best option as a Tomondo companion. For example, the combined model ranked someone with a chronic illness first, followed by a family member, and then a friend. However, among the individual group models, ICDs and promotores both ranked attending with a family member first (ICD = 0.45 and P = 0.46) and placed less weight on attending with someone else with a chronic illness (ICD = 0.36 and P  = 0.37). However, the FM model placed the most weight on attending with someone who has a chronic illness first (FM = 0.55), followed by attending with a family member (FM = 0.33). Across all three models, attending with a friend was ranked lowest (C = 0.16).

### 3.3 Priming participants for an intervention

**Figure 2:**
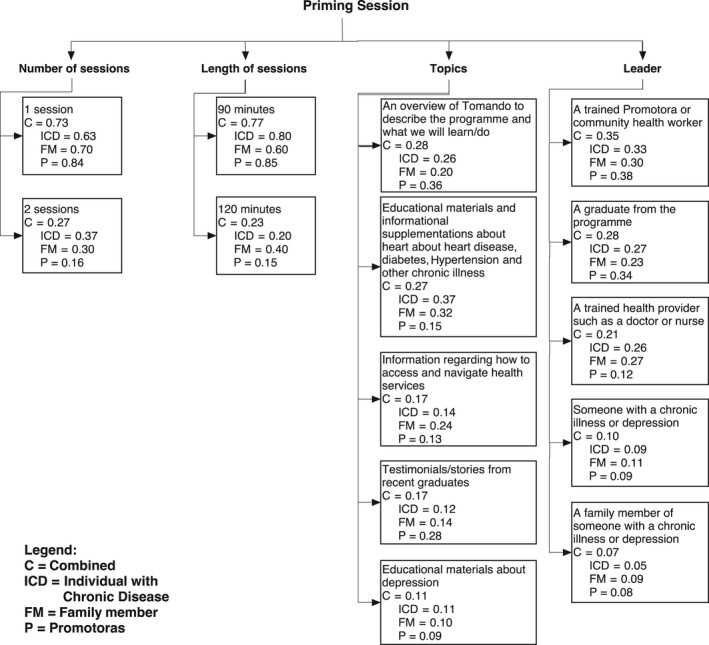
Identifying elements of the priming session

The Stakeholder Values Questionnaire also sought to further elucidate information on potential additions to the intervention, including a ‘priming’ or introductory component. Specifically, participants were asked to weigh options to inform the development of a potential introductory session(s), including the number, length, content and who should lead the session. Ranks and associated weights for each option are presented in Figure 2.

Overall, there was consensus across models regarding the number of sessions that would be optimal to prepare individuals for participating in a CDSMP programme, with one session as the preferred option (C = 0.73). Consensus also existed across groups regarding the length of these sessions. While 90‐minute sessions were ranked as the preferred option among all groups (C = 0.77), family members placed less weight on this option (FM = 0.60) than ICDs (ICD = 0.80) and promotoras (P = 0.85).

Moreover, participants were asked to rank potential topics that might be covered in a priming session. Variation existed between groups in both the rank ordering of topics as well as their weight. Specifically, the promotora model placed more weight on having an overview of the programme (P = 0.36) that described key features of Tomando and its elements, followed by testimonials from recent graduates (P = 0.28). Less emphasis was placed on educational materials and information (P = 0.15). In contrast, both ICDs and FMs ranked having educational materials and informational supplements about heart disease, diabetes, hypertension and other chronic illness as their primary choice (ICD = 0.37 and FM = 0.32). All groups placed considerably less weight on educational materials about depression (C = 0.11).

Participants were also asked who should lead priming sessions. Consensus existed across all groups, ranking promotoras/community health workers as the optimal choice (C = 0.35). However, variation existed in terms of the other options, with both the ICD and promotora models ranking a graduate from the programme second (ICD = 0.27 and P = 0.34) and a trained health‐care provider third (ICD = 0.26 and P = 0.12). In contrast, a trained health‐care provider was ranked second in the FM model (FM = 0.27).

### 3.4 Sustaining positive outcomes

**Figure 3:**
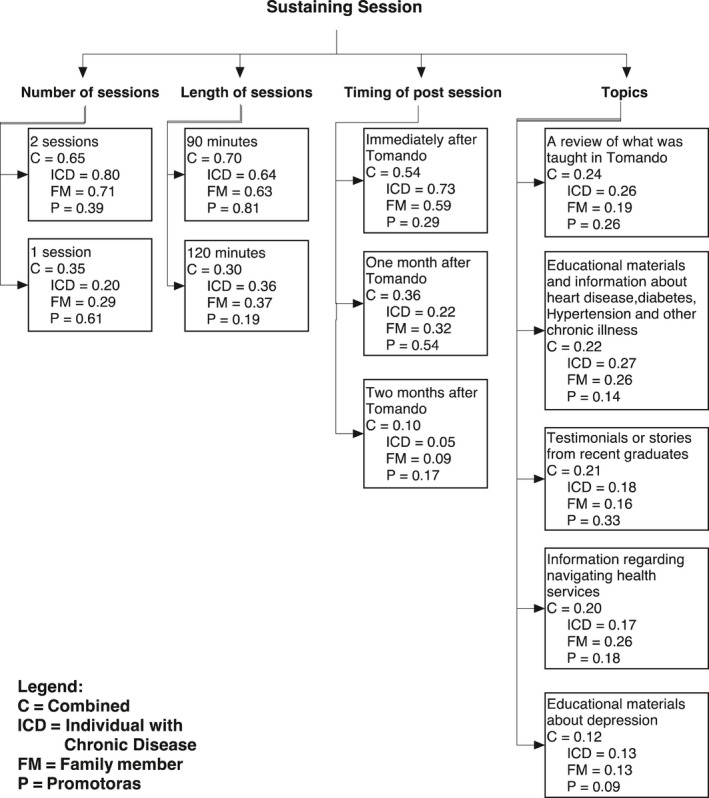
Identifying elements of the sustaining session

The Stakeholder Values Questionnaire also elicited information regarding elements that would help individuals sustain successful management of their condition after completing the Tomando programme. Thus, participants were asked to share their opinions regarding the addition of a sustaining session(s). Again, participants were asked to weigh options to inform the development of a potential follow‐up session(s), including the number, length, content and leader of such a session(s). Ranks and associated weights for each option are presented in Figure 3.

ICDs and FMs clearly ranked having two sustaining sessions as the ideal number (ICD = 0.80 and FM = 0.71), aligning with the idea of desires for assuring optimal learning and benefit through the added sustaining sessions. In contrast, promotoras placed greater weight on having one sustaining session (P = 0.61). All participants ranked having 90‐minute sustaining sessions over 120‐minute sessions (C = 0.70). Participants also answered a series of questions regarding the timing of sustaining sessions. The combined model ranked having the sustaining sessions immediately after the programme as first (C = 0.54), followed by one month after (C = 0.36) and two months after (C = 0.10). However, variation existed across models. ICDs and FMs placed the greatest weight on having the sessions immediately (ICD = 0.73 and FM = 0.59), followed by one month after (ICD = 0.22 and FM = 0.32). Additionally, the strength of this difference among ICDs is noteworthy (0.73 compared to 0.22 one month after and 0.05 two months after Tomando). In contrast, promotores ranked beginning the sustaining sessions one month after Tomando as their first choice (P = 0.54) and immediately after Tomando as their second choice (P = 0.29).

Participants also guided the content that would be presented in a potential sustaining session. Specifically, participants were asked to rank the important elements of a sustaining session, including a review of Tomando materials, additional educational materials regarding chronic illnesses, graduate testimonials, information regarding navigating the health system and educational materials about depression. Overall, participants ranked a review of Tomando first (C = 0.24), followed by the delivery of supplemental educational materials and information on chronic diseases (C = 0.22), testimonials from Tomando graduates, information about accessing and navigating health services (C = 0.20) and educational materials about depression (C = 0.12). Variation across models was noted. Both ICDs and FMs ranked educational components focused on chronic illness first (ICD = 0.27 and FM = 0.26), compared with promotores who ranked having testimonials or stories from recent graduates first (P = 0.33). Both promotores and ICDs ranked a review of Tomando as second (P = 0.26 and ICD = 0.26), while FMs ranked receiving information on how to access services second (FM = 0.26). This option was ranked fourth by both ICDs and promotores (ICD = 0.17 and P = 0.18). Across groups, receiving additional educational materials on depression was ranked last (C = 0.12).

## 4 DISCUSSION

This study illustrates the utility of employing a novel approach for assessing the needs and preferences of multiple stakeholders for ultimately informing the augmentation and implementation of an evidence‐based chronic disease programme for Latinos affected by chronic illness and co‐occurring minor depression. While, overall, the core elements of the Tomando programme were well received, findings from the formative research phase^29^ and results of the present study demonstrate that additional adaptations and tailoring the programme for the specific target population may enhance outcomes. Employing AHP allowed for a detailed and rigorous exploration of these potential additions, while also ensuring consideration of the critical components of the Tomando programme.

AHP stands out from other evaluation and planning techniques in that it relies heavily on the population being affected by a problem or decision and allows their preferences and values to be translated into a scaled ranking, leading to the data being ‘invariant to politics and behaviour’.^11^ Thus, AHP allowed for a rigorous approach to programme adaptation by facilitating the comparison of priorities across stakeholder groups and as a combined group, thereby reducing bias in the reporting of overall group decisions and allowing the research team to elucidate varying preferences across subgroups.

When implementing EBIs, it is vital to ensure local acceptability in regard to the target population and their needs and potential challenges. However, it is also fundamental to ensure fidelity and maintain the original elements of the EBI that have been proven to be effective.^35^, ^36^ Researchers must often balance the demands of holding true to a validated programme while also meeting the needs of the local population, especially as culture and language influence perceptions of health, health behaviours and access to resources. Utilizing the stakeholder‐driven methods employed in this study allowed the research team to confirm the critical importance of the core Tomando elements, while also considering emergent stakeholder needs through the formative research stage to be assessed further, allowing their relative importance to drive decision‐making.

Careful attention was paid to which stakeholder groups placed emphasis on which of the various factors considered. The literature suggests that discordant patient and provider preferences for health‐care intervention attributes is common, and researchers have called for stronger assessment of the heterogeneity of responses across patient‐provider groups.^37^ Thus, this research is novel in that it elicited critical responses from multiple key end‐user populations of a potential health intervention—the individuals living with the chronic disease, their families and the promotoras who would deliver the intervention—with the goal of implementing a better‐suited intervention. AHP allowed the Research and Implementation Team, which was comprised of a diverse group of researchers, community partners active in the local Latino community and members of a community advisory board brought together to inform and oversee the research, to tease out concordant and discordant preferences. The resulting data, and the examination across and within each subgroup, allow for deeper understanding of findings.

In certain instances, differences emerged by subgroup, likely differences were based on background or lived experience. For example, caregivers focused on including nutrition content in the programme, which is often an important aspect in caregiving.^38^ ICDs were more focused on stress management and dealing with difficult emotions, potentially highlighting the daily struggle with the effects of chronic conditions.^39^, ^40^ Promotoras tended to focus on traditional best practices for training and education. Thus, when decisions were made, the Research and Implementation Team teased out each individual group's preferences and assessed the meaning behind the value. For example, when asked about the type of content needed for the augmented programme, promotoras placed weight on educational materials, a choice that generally aligns with their training and expertise.^41^ One of the greatest contributions of promotores is their knowledge of the community they serve and its specific needs.^41^, ^42^ The prioritization of the informational and educational materials for managing chronic illness by promotores reflects their nuanced perspective. In contrast, ICDs and FMs ranked skill‐building as most important for programme content, which may be more immediately beneficial to them, as receiving the skills to manage a chronic condition may be considered more advantageous than learning about that condition. These contrasting prioritizations demonstrate the merit of both education and skill‐building, while highlighting the need for certain elements based on the insight of key end‐user groups. Ultimately, these contrasting priorities resulted in the addition of role‐playing activities that would put into practice both skill‐building content as well as the informational and educational materials that allowed for experiential learning.

The application of AHP also allowed for the identification of congruence between groups, as well as the nuanced differences. For example, all groups prioritized the addition of sessions to the original Tomando model, though variation existed in the desired content of these sessions. For example, the promotora model ranked an overview of Tomando to introduce the programme and its elements first followed by testimonials from programme graduates. This is likely a result of promotoras' experiences with the motivational influence that can come from hearing the experiences of peers who have participated in the programme. However, both ICDs and FMs ranked supplemental educational materials and informational on chronic illnesses first, focusing on the more tangible and practical information. Such differences may reflect the differential experiences and needs of each subgroup, and AHP allows researchers to tease out these differences. Promotoras may place a higher value on orientation towards the programme in order to enhance participant understanding and engagement, while ICDs and FMs may be more content‐focused, driven by desires for educational materials that could be directly implemented into participants' daily lives. Ultimately, the Research and Implementation Team negotiated these contrasting prioritizations, following promotores' suggestions for orientation and testimonials in the priming sessions based on their expertise and experience with community‐based Latino health programme, while ensuring the educational materials desired by ICDs and FMs were delivered in the sustaining sessions. This approach also allowed for maintenance of programme fidelity (educational materials are not part of the original Tomando) and comparability of outcomes between the original and augmented Tomando programmes.

Utilization of AHP methods also elucidated the variations in stakeholder needs and priorities regarding the sustaining sessions, with promotoras preferring a single sustaining session one month after completion of the Tomando programme, while ICDs and FMs ranked two sustaining sessions immediately following Tomando first. This prioritization may suggest the need to balance individual's participation in Tomando with other daily demands including work, childcare, lack of transportation and household responsibilities.^43^ Although ICDs and FMs indicated preference for sustaining sessions immediately after the final original programme session to promote sustained, positive outcomes, promotoras indicated that one month after the programme would be better from their perspective. Taking these prioritizations into consideration alongside relevant health education literature, decisions were made to prioritize promotoras' assessments, while also negotiating the expressed desires of ICDs and FMs by adding two sustaining sessions, including the requested educational materials, and the mailing of *metas* (goals) written by participants during the programme. These *metas* served as motivational boosters for participants in order to enhance long‐term, positive outcomes.^44^

Finally, while participants overwhelmingly agreed that attending Tomando with a partner would be best, whom that person should be varied across groups. Through the AHP process, researchers found that ICDs desired a companion that was a family member, with promotoras agreeing. In contrast, FMs prioritized attending with another person who had a chronic illness. Through personal experience, it is likely that ICDs and promotores see the integral role that family members play in disease management, while family members may feel that a person with a chronic illness could better relate to and understand an ICD's experience. However, it is also important to note that these categories are not mutually exclusive as some family members in this study also had a chronic illness.

Despite contrasting priorities, through the application of AHP, this study was able to clarify the priority needs of the target population and better adapt the content to local needs and preferences. The data generated allowed the study team to identify and negotiate varying, congruent and contrasting needs and priorities across the three stakeholder groups, providing valuable quantitative insight to inform augmentation.

This research demonstrates the utility of AHP for future health education and health promotion‐related research. Accordingly, the authors recommend the incorporation of AHP methods into the research process, particularly the adaptation of validated EBIs for local settings, as such an approach can enhance the feasibility of resultant programmes as well as increase their adoption by the local community through the incorporation of stakeholder voices. The use of AHP in this study allowed researchers insight into various stakeholder groups, their priorities and needs, and the value they place on various aspects of the validated EBI. Moreover, through AHP, researchers were able to compare and contrast these different stakeholder groups' feedback in a rigorous, quantitative manner, a level of detail that is often difficult to elucidate. Through this approach, this study was able to breakdown the various elements of *Tomando* in order to focus on how to best tailor the programme to the needs of multiple stakeholder groups. Further, the use of AHP maintains the potential to enhance community‐based, participatory research methods through its rigorous methodology and stakeholder engagement, allowing for stronger partnerships.

### 4.1 Limitations

This study models an innovative technique for the adaptation of culturally tailored interventions. However, the majority of participants were female (80%). Additionally, nearly a quarter of ICDs were unemployed. This may have influenced views about timing, the length and number of sessions in the intervention and family member participation. Additional analysis with a larger sample size is recommended for future studies.

## 5 CONCLUSION

The varying responses presented by participants overall and across the three different stakeholder groups provide critical insight into the components of an EBI that may resonate with different participant groups. Understanding these varying needs and perspectives allows investigators to select the elements that best reach the target audience and their needs and are more likely to ensure success of an intervention. This is where the value of AHP is most evident. As a research tool, AHP allows investigators to individually tease out the priorities and preferences for each stakeholder group, while also creating a combined score in a mathematically sound fashion. Together, these scores (each individual score and the overall combined score) help to drive decision‐making, while also reducing investigator bias in the decision‐making process. Thus, the implications of utilizing AHP in a health‐focused context are enhanced local adaptation for target populations and their specific needs as well as considerations for how different stakeholder groups' needs can be negotiated and balanced with respect to each other. In the case of the present study, findings were used to adapt an EBI to local needs by adding both priming and sustaining sessions to the Tomando programme, with content focused on the priorities assessed through AHP analysis and delivery methods adapted according to the insights gained from this process. These changes ultimately resulted in the implementation of a programme that had a significantly greater impact on the reduction of minor depression among individuals with co‐morbid chronic disease and minor depression than the standard EBI.^32^, ^45^

## CONFLICT OF INTEREST

The authors certify that they have NO affiliations with or involvement in any organization or entity with any financial interest or non‐financial interest in the subject matter or materials discussed in this manuscript. There are no conflicts to report.

## Data Availability

The data that support the findings of this study are available from the corresponding author upon reasonable request.
